# Superconductivity in Crystallographically Disordered LaHg_6.4_

**DOI:** 10.1021/acs.inorgchem.2c01987

**Published:** 2022-09-02

**Authors:** Yurii Prots, Mitja Krnel, Yuri Grin, Eteri Svanidze

**Affiliations:** Max-Planck-Institut für Chemische Physik fester Stoffe, Nothnitzer Str. 40, Dresden01187, Germany

## Abstract

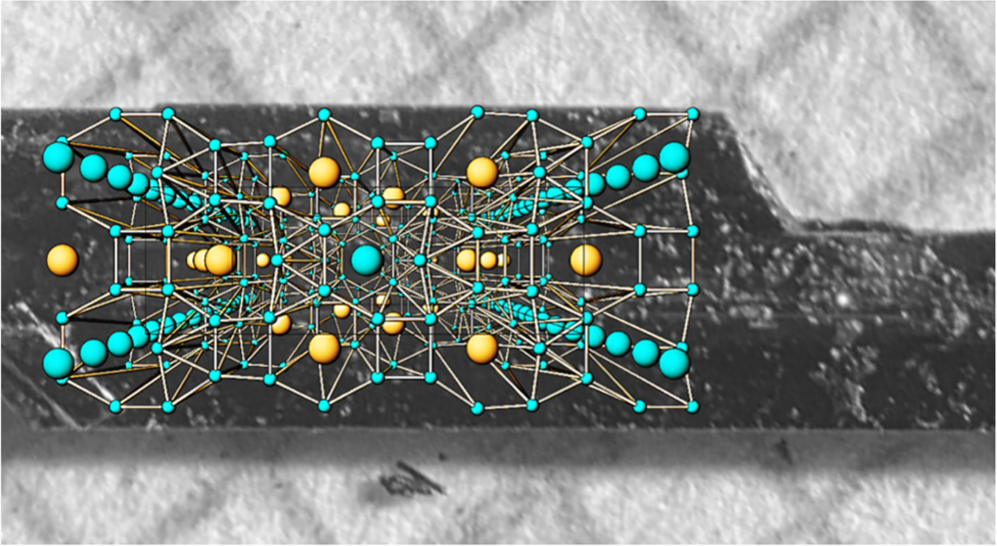

The influence of structural disorder on superconductivity is not yet fully understood. A concurrent examination of crystallographic and physical properties of LaHg_6.4_ reveals that this material enters a superconducting state below *T*_c_ = 2.4 K while showing crystallographic disorder in one dimension. Lanthanum mercuride, which crystallizes in a new structure type (space group **Cmcm*, a* = 9.779(2) Å, *b* = 28.891(4) Å, *c* = 5.0012(8) Å, *Z* = 8), has remained out of reach for nearly 50 years. In this crystal structure, strong disorder is present in the channels that propagate along the [001] direction. By implementing a combination of cutting-edge synthesis and characterization techniques, we were able to circumvent the complexity associated with the low formation temperature and chemical reactivity of this substance and study the superconductivity of LaHg_6.4_ in detail.

## Abstract 2

Superconductivity in crystallographically disordered LaHg_6.4_ is observed. A comprehensive analysis of the crystallographic properties of this material reveals strong disorder in channels, propagating along the *c*-axis. The bulk superconductivity of LaHg_6.4_ below *T*_c_ = 2.4 K, observed in several types of measurements, appears to be conventional in nature. This in-depth investigation from both chemical and physical perspectives has only been possible due to a set of specialized experimental tools and techniques.

## Introduction

It is well known that the chemical and physical properties of solid-state materials are deeply interrelated.^1−3^ In particular, changes in the crystal lattice and the resultant structural disorder are frequently used as tuning parameters to change the ground state of a given material. Although typically, the lack of translational symmetry prohibits the appearance of superconductivity, the coexistence of the latter with structural disorder has been observed in some two- and three-dimensional materials.^4−11^ For one-dimensional compounds, the effect of disorder on both bulk and topological superconductivity is not yet well-understood.^12−15^

Mercury-based compounds (amalgams) have received a considerable amount of attention in the chemical community—these materials often host complex crystallographic arrangements^16−27^ and display peculiar bonding features.^22,28−34^ As a result, some of these materials show peculiar superconducting characteristics,^21,32,35−47^ including high critical temperatures, which can be enhanced even further by the application of pressure. Some mercury-containing materials were even suggested to exhibit topologically nontrivial states.^48−54^ Moreover, peculiar magnetic correlations, brought on by the mixing of d and f orbitals, have been observed in several solid-state compounds.^23,51−60^ Additionally, the application potential of some mercury-based materials has been discussed.^23,61^ However, it is well-known that much care must be taken during the synthesis, handling, and characterization of mercury-based compounds, in particular rare earth metals’ mercurides—from toxicity concerns to their high chemical reactivity—as these systems pose several experimental challenges.^27,62−64^ The complexity of work on mercury-based materials is reflected in a low number of systems that have been discovered so far. Furthermore, detailed investigations of chemical and physical properties are often missing even for binary and ternary compounds that are already known. In the La–Hg system, the existence of LaHg,^17^ LaHg_2_,^17^ LaHg_3_,^65^ La_11_Hg_45_,^64^ La_13_Hg_58_,^65^ and LaHg_6∼_^65^ has been reported. However, an in-depth analysis of their properties remains to be carried out.

## Results and Discussion

**Figure 1:**
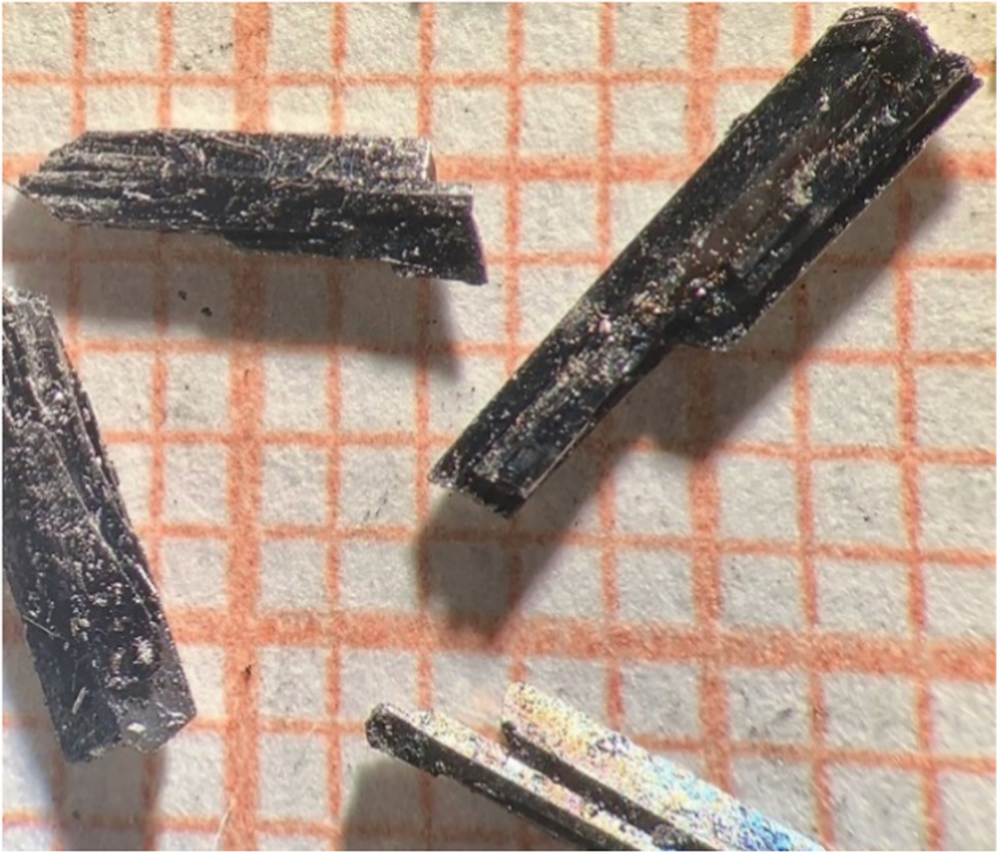
Single crystals of LaHg_6.4_. Minute exposure to air results in an immediate degradation of the sample, as evidenced by the rough surface of the single crystals and microscopic mercury droplets, appearing on their surface. The smallest squares have a dimension of 1 mm × 1 mm.

The newly rediscovered lanthanum mercuride LaHg_6.4_ is the most mercury-rich phase in the La–Hg system. While the first report regarding the existence of the “LaHg_6∼_” compound suggested orthorhombic crystal structure and lattice parameters in 1976,^65^ the comprehensive structural characterization of LaHg_6.4_ has been missing for nearly 50 years. This can be understood by the experiential difficulty of mercury-based materials—in addition to high X-ray absorption, the LaHg_6.4_ compound is extremely air-sensitive, decomposing immediately even after a few second of air exposure. We have therefore prepared and characterized mm-sized single crystals of LaHg_6.4_ (Figure 1) in a protective environment using several dedicated experimental techniques.

**Figure 2:**
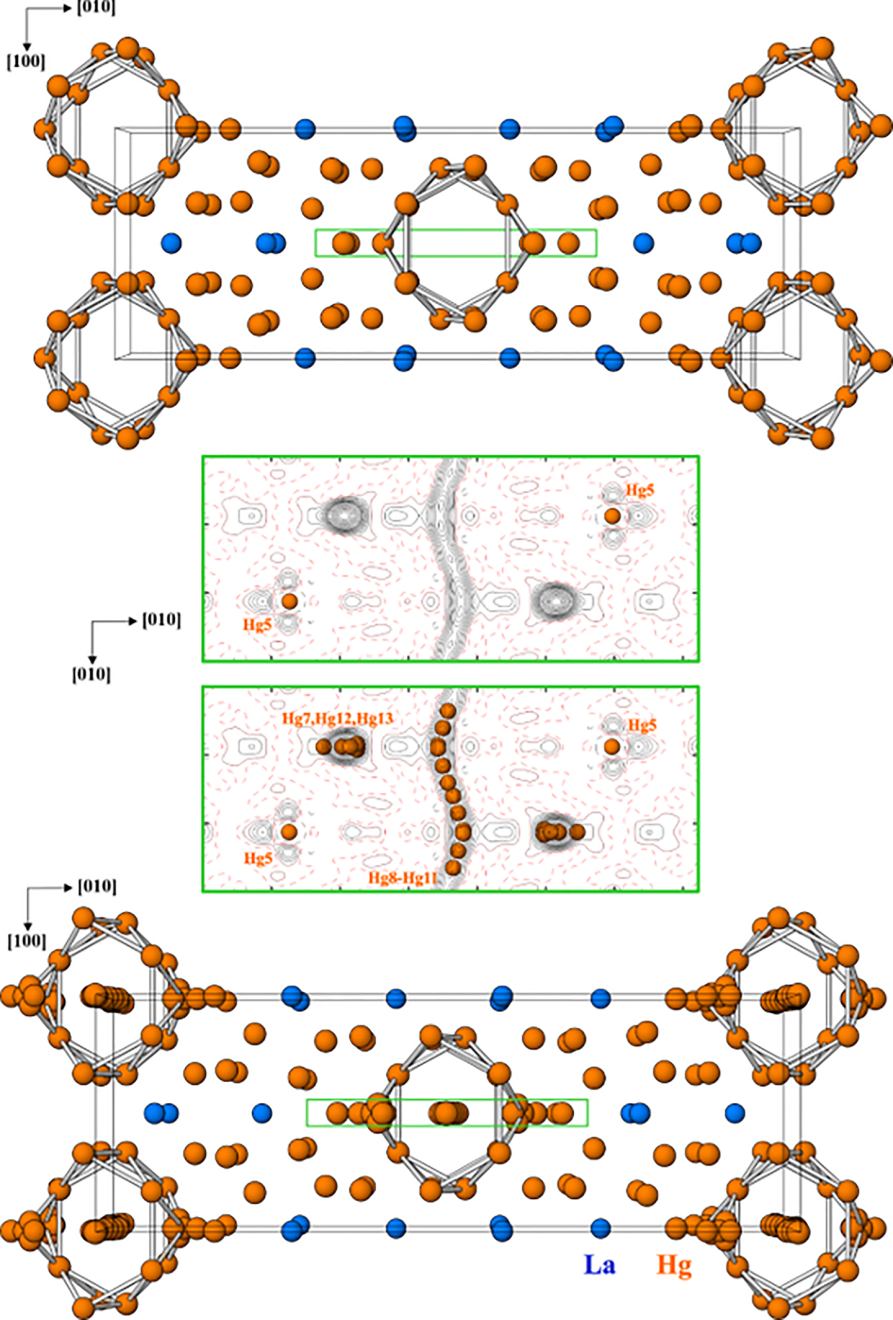
Crystal structure of LaHg_6.4_: (top) basic atomic arrangement with channels running along the [001] direction with the main axes [00*z*] and [^1^/_2_ ^1^/_2_ *z*]; (middle) distribution of the difference electron density within and around the channels (green region in the top and bottom panels) with mercury split positions used for its description; and (bottom) a perspective view of the complete crystal structure along [001].

The crystal structure was conclusively established based on the single-crystal X-ray diffraction experiment (see Tables S1–S3 for the corresponding crystallographic information). The diffraction data were indexed using orthorhombic lattice parameters *a* = 9.779(2) Å, *b* = 28.891(4) Å, and *c* = 5.0012(8) Å (close to those reported earlier^65^), and the extinction conditions agreed well with the C-centered lattice indicated in ref (65). Application of charge-flipping techniques allowed to determine the basic atomic arrangement (Figure 2, top), implying enlarged atomic displacement parameters for selected mercury atoms (Hg5, Hg7) and channels within the mercury sublattice formed by Hg2, Hg6, and Hg7 and running along the [001] direction with the main axes [00*z*] and [^1^/_2_ ^1^/_2_ *z*]. Calculation of residual electron density with only La and Hg atoms of the basic atomic arrangement reveals quasi-continuous density distribution along the channels’ axes (Figure 2, middle). For its appropriate description, additional partially occupied mercury positions were used (Hg10, Hg11). The atomic chain that fills up the channel is obviously incommensurate with respect to the remaining structure. Moreover, due to the large distance between the channels, their occupation seems to not be synchronized. This leads to the disorder around the Hg5 and Hg7 atoms, located at the channels’ walls, and split positions have to be used to describe the local order in this region. While the split position Hg5 is completely occupied, the sum of occupancies of the Hg7, Hg8, and Hg9 positions is not unity, i.e., the noncommensurable occupation of the channels causes “breathing” within the channel wall at some places. Further signs of local violations of the translational symmetry can be recognized in the strong anisotropy of the atomic displacement of the Hg6 and La1 positions, which are located in the surrounding of the channels. This results in deviations from the previously suggested 1:6 stoichiometry,^65^ yielding composition LaHg_6.4_, i.e., LaHg_6–*x*+*y*_. Partial incommensurability can sometimes be described by means of modulations. In the case of LaHg_6.4_, no satellite reflections were observed in the single-crystal X-ray diffraction experiments. Thus, the establishment of the modulation vector was not possible.

The majority of the Hg–Hg distances vary as 2.88 Å ≤ *d*_Hg–Hg_ ≤ 3.39 Å, similar to what is observed in elemental Hg with *d*_Hg–Hg_ = 2.99–3.46 Å.^73^ A comparison of the latter values with the lattice parameter *c* = 5.0012(8) Å of LaHg_6.4_ indicates that it is not possible to localize 2 Hg species along this direction within one lattice parameter, as required by the symmetry of the space group **Cmcm** or its noncentrosymmetric variants. Thus, local symmetry breaking is present. Attempts to refine the data using noncentrosymmetric groups did not yield lower residual factors and did not allow to model ordering in the channels.

Interestingly, the short lanthanum–lanthanum contacts appear to be absent, with the shortest *d*_La–La_ = 4.93 Å being rather large. In comparison, those observed in elemental lanthanum are *d*_La–La_ ∼ 3.76 Å. For the lanthanum atoms, two crystallographic positions exist: La1 is coordinated by 14 Hg atoms in the form of a bicapped hexagonal prism, while La2 is coordinated by 13 Hg atoms. The coordination of La2 can be described as a modified cuboctahedron, where one of the apexes (Hg) is replaced by two Hg atoms. For a polyhedral representation of the structure, see Figures 1 and 4 in ref (66). In comparison, in mercury-poorer La_11_Hg_45_, lanthanum atoms have regular cuboctahedra as coordination polyhedra.^16^

**Figure 3:**
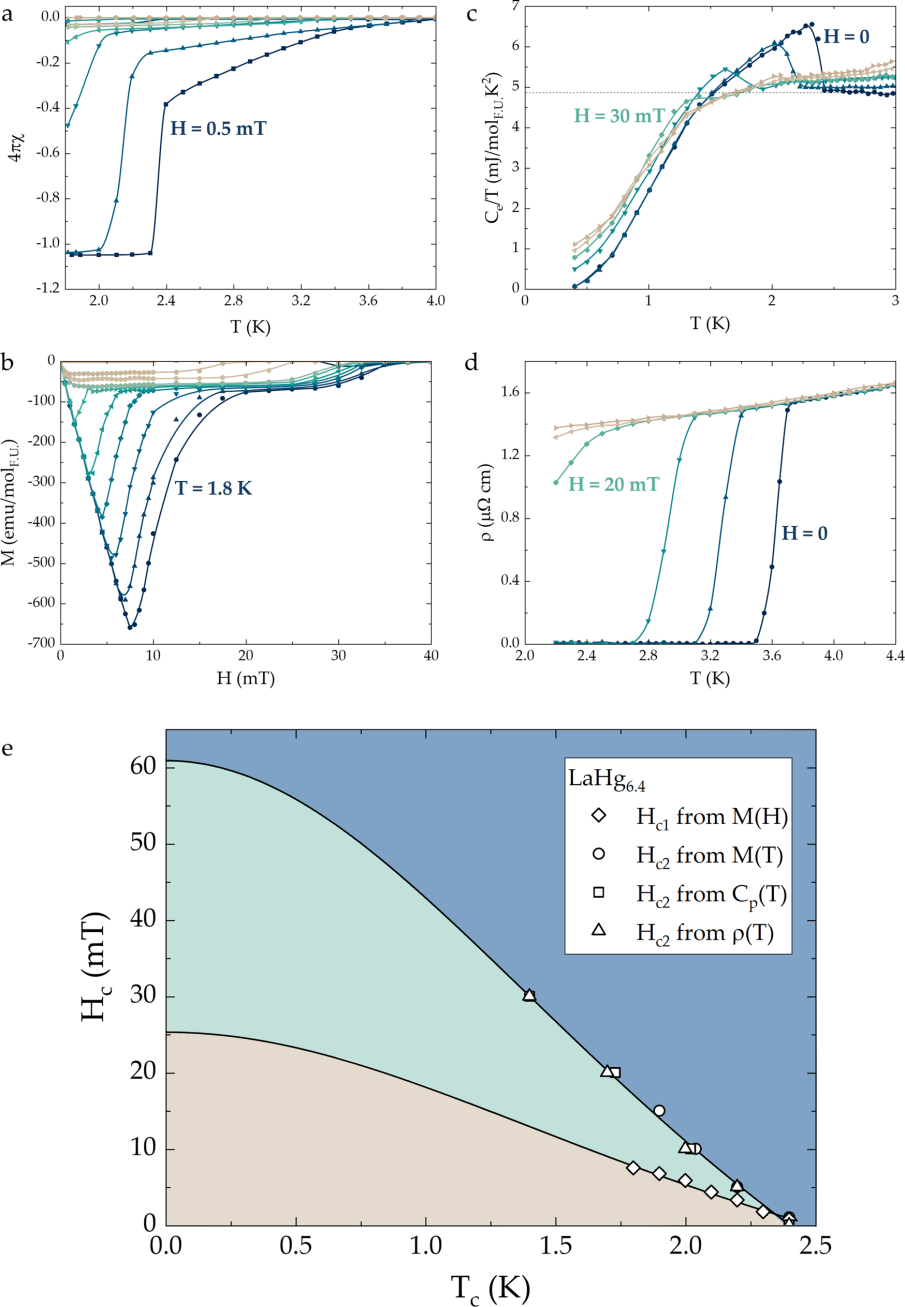
Superconducting properties of LaHg_6.4_. Magnetization as a function of (a) temperature and (b) magnetic field. Temperature-dependent (c) specific heat and (d) resistivity data in various applied magnetic fields. (e) The phase diagram for LaHg_6.4_ has three regions typical for a type-II superconductor.

The first indication of superconductivity in LaHg_6.4_ is given by a sharp diamagnetic transition at *T*_c_ = 2.4 K (Figure 3a), observed in zero-field-cooled magnetic susceptibility data. Given that the superconducting volume fraction is over 100%, magnetization data indicate bulk superconductivity of the studied sample, which is further confirmed by specific heat and resistivity data, shown below. The crystals of LaHg_6.4_, which have a rectangular rod-like morphology (see Figure 1), were oriented with the *c*-axis, i.e., the longer side of the crystal, parallel to the direction of the applied magnetic field. A slight enhancement of the Meissner fraction above the value of 1 can be explained by microscopic inclusions of Hg in the LaHg_6.4_ crystals. The shape of magnetic isotherms (Figure 3b) is consistent with type-II superconductivity in this compound. The presence of residual mercury^67,68^ on the surface as well as inside the LaHg_6.4_ crystals is also evident from the secondary transitions, observed in the temperature- and field-dependent magnetization data around *T* = 4 K (Figure 3a) and *H* = 30 mT (Figure 3b), respectively. The bulk superconductivity of the LaHg_6.4_ compound is confirmed by the anomaly in the specific heat data, as shown in Figure 3c. The value of the electronic specific heat coefficient γ = 4.8 mJ mol_F.U._^–1^ K^–2^, while the size of the specific heat jump Δ*C*_e_/γ*_n_T*_c_ = 0.75 is significantly less than the expected BCS value (Δ*C*_e_/γ*_n_T*_c_ = 1.43). The latter can likely be attributed to the difficulties of background subtraction for samples with a small mass (see Materials and Methods section for further details). The Debye temperature θ_D_ = 424 K is used to estimate the magnitude of the electron–phonon coupling λ_e–p_ = 0.43, consistent with the conventional superconductivity of LaHg_6.4_.

The superconductivity of LaHg_6.4_ can be gradually suppressed by the application of a magnetic field, as observed from magnetization, specific heat, and resistivity data. The resultant *H*–*T* phase diagram (Figure 3e) is typical of that of a type-II superconductor. The superconducting state is observed below *T*_c_ and lower critical field *H*_c1_(0) = 25 mT. The region between the superconducting and normal states, also known as the mixed state, occurs below *T*_c_ and *H*_c2_(0) = 61 mT. The Ginzburg–Landau parameter κ(0) = 3.2 > 1/√2 is in agreement with LaHg_6.4_ being a type-II superconductor.

## Conclusions

In this work, we present the discovery and characterization of LaHg_6.4_—a quasi-one-dimensional (1D) crystallographically disordered compound that enters the superconducting state below *T*_c_ = 2.4 K. This material has remained out of reach for nearly 50 years, as a result of experimental challenges of the work on mercury-containing compounds (amalgams). Using a number of cutting-edge synthesis and characterization tools, we were able to synthesize mm-sized single crystals of LaHg_6.4_, which shows bulk superconductivity below *T*_c_ = 2.4 K. Further investigations of the electronic structure of LaHg_6.4_ and isotypic phases are expected to shed more light on the relation between dimensionality and disorder in this peculiar material.

## Materials and Methods

All sample preparation and handling were performed in a specialized laboratory, equipped with an argon-filled glovebox system (MBraun, *p*(H_2_O/O_2_) < 0.1 ppm).^69^ Single crystals of LaHg_6.4_ were synthesized from La (pieces, Ames Laboratory, >99.9%) and Hg (ChemPur, 99.999%) using the self-flux method. The La chunks and Hg droplets, mixed in the 5:95 ratio, were sealed in Ta tubes under an Ar atmosphere (Figure S1a,b). The volume of the Ta tubes, chosen so as to exclude Hg loss, was ∼2 cm^3^ for a total sample mass of 1 g. The sealed Ta tubes were heated to 500 °C and then slowly cooled to room temperature over a period of 10 days. Excess Hg flux was decanted at room temperature via centrifugation in custom-made crucibles shown in Figure S1b. Residual mercury could not be completely removed from the surface of the crystals. The resultant crystals had silver luster and needle- or slab-like morphology, with some examples shown in Figure S1c. Despite strong crystallographic disorder, the value of the residual resistivity ratio is rather large, RRR = 63. Similar to the other mercury-based binary compounds,^19,57,70^ the LaHg_6.4_ phase exhibited extreme air and moisture sensitivity, resulting in immediate decomposition even after a few seconds of exposure to air. Additionally, the LaHg_6.4_ crystals were rather fragile, breaking easily when touched by tweezers.

Powder X-ray diffraction was performed on a Huber G670 Image plate Guinier camera with a Ge monochromator (CuKα_1_, λ = 1.54056 Å). Phase identification was done using WinXPow software.^71^ The lattice parameters were determined by a least-squares refinement using the peak positions, extracted by profile fitting (WinCSD software^72^). Small single crystals with a size of ∼50 μm were suitable for single-crystal diffraction experiments. Single-crystal diffraction data were collected using a Rigaku AFC7 diffractometer, equipped with a Saturn 724+ CCD detector and a MoKα radiation source (λ = 0.71073 Å). WinCSD software^72^ was used for crystallographic calculations. The crystallographic information is provided in Tables S1–S3.

**Figure 4:**
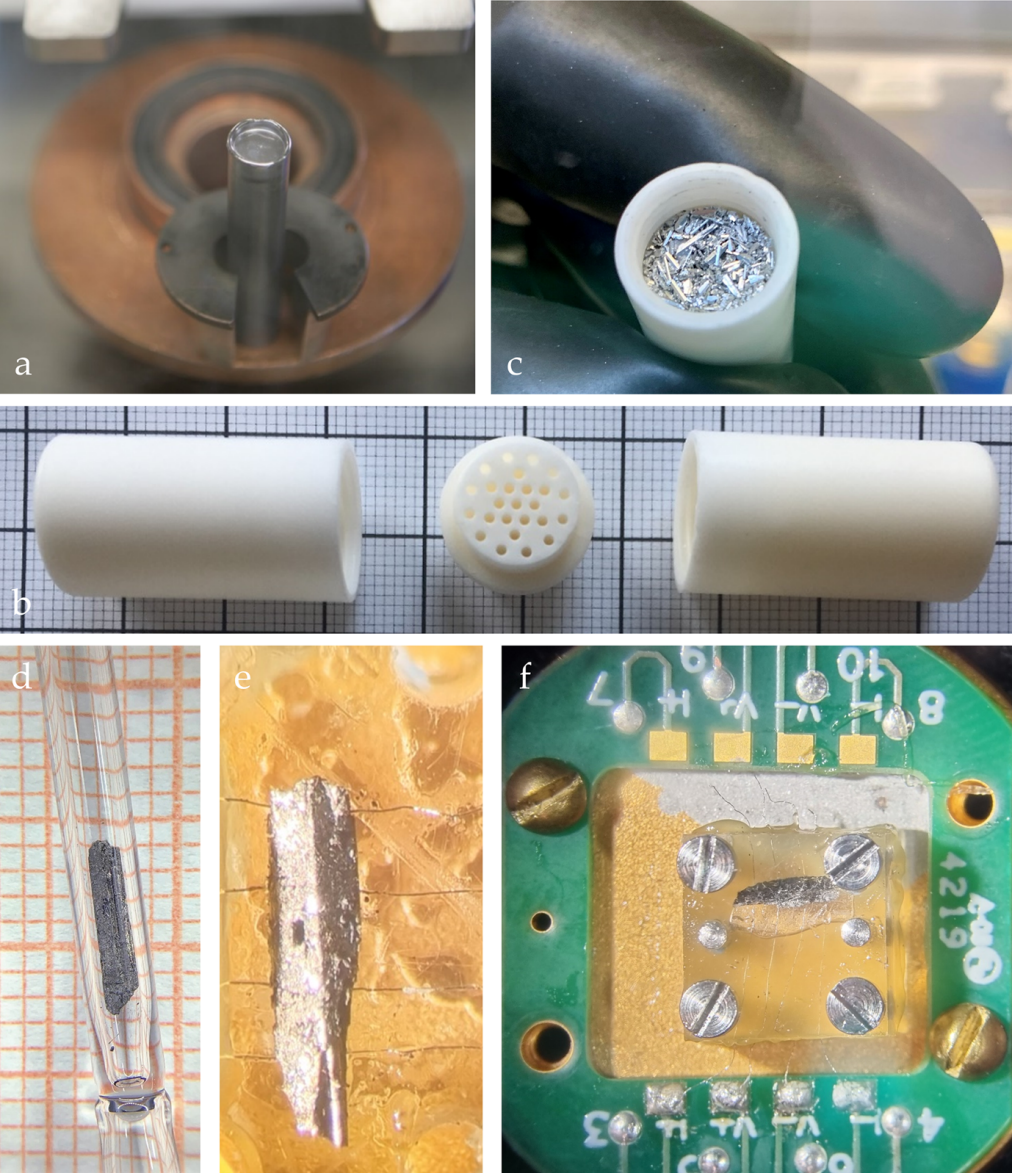
Synthesis and characterization of LaHg_6.4_. (a) Sealed Ta ampoules contain custom-made alumina crucibles, shown in (b). (c) The residual mercury is easily decanted. The resultant crystals have needle- or slab-like morphology. (d) To protect samples from oxidation, single crystals are sealed under argon inside a quartz capillary. (e, f) A cell designed for measuring the resistivity of air-sensitive samples—wires are held in place by a Plexiglas platform, and the sample is surrounded by vacuum grease.

The magnetic properties were studied using a Quantum Design (QD) Magnetic Property Measurement System for the temperature range from *T* = 1.8 to 300 K and for applied magnetic fields up to *H* = 7 T. Individual crystals were sealed inside glass tubes, both to protect the sample from oxidation and to ensure sample orientation, with an example shown in Figure 4d. Temperature- and field-dependent magnetization measurements were performed with a magnetic field applied parallel to the *c*-axis, eliminating the need for demagnetization correction. The Meissner fraction was determined from zero-field-cooled data as 4πχ, using the calculated density of LaHg_6.4_ of 13.44 g cm^–3^. The value of the Meissner fraction exceeding 1 can be attributed to either (i) excess elemental mercury inside or on the surface of the crystals or (ii) deviations of the sample shape from the ideal cylinder geometry. The former is supported by the observation of a feature associated with the freezing of elemental mercury in the temperature-dependent resistivity data (see Figure S1). The values of *H*_c1_ were extracted from magnetic isotherms, shown in Figure 3b, as the field at which the *M*(*H*) curves deviate from the line with the initial slope of the *M*(*H*) curve. The values of *H*_c2_ were extracted from temperature-dependent magnetization, specific heat, and resistivity data. For the latter, a constant temperature offset was used to account for the poor thermal coupling of the sample and the platform (see below).

The specific heat data were collected on a QD Physical Property Measurement System (PPMS) in the temperature range from *T* = 0.4 to 10 K for magnetic fields up to *H* = 9 T. Given the high air sensitivity of the LaHg_6.4_ crystals, a large amount of grease was used to protect the sample. This, together with the small size of the signal produced by the LaHg_6.4_ crystal, can sometimes lead to a large background contribution to the specific heat. As a result, it is possible that the magnitude of the specific heat jump is underestimated.

For resistivity measurements, it was noticed that silver paint or glues react with the surface of the LaHg_6.4_ crystals, producing a thin layer of mercury. In this case, the resistivity measurements are dominated by the properties of mercury. To address this challenge, a special cell was designed—see Figure 4e,f. The handling of the cell was done inside a glovebox. The cell is composed of two sheets of Plexiglas, held together by screws, with platinum wires pressed on top of the sample. A layer of grease was used to protect the sample from oxidation. The superconducting transition observed in resistivity was offset by approximately 1 K from that observed in magnetization and specific heat data. This can either be attributed to (i) the thermal lag in coupling between the sample and the platform or (ii) the pressure effect, induced by the plates. The inherent reason behind this discrepancy is still being investigated.

## Supporting Information Available

Crystallographic data, together with electrical resistivity measurements (PDF)
