# Supplementary material for: Superconductivity in Crystallographically Disordered LaHg6.4
Source: Inorg Chem. 2022 Sep 2;61(39):15444–51. doi: 10.1021/acs.inorgchem.2c01987 (PMC9533302; doi:10.1021/acs.inorgchem.2c01987)
Supplement: Supplementary file 1 — ic2c01987_si_001.pdf [file ic2c01987_si_001.pdf]

Superconductivity in crystallographically disordered $LaHg_{6.4}$

Yurii Prots[1], Mitja Krnel[1], Yuri Grin[1], Eteri Svanidze[1*†]

[1]*Max-Planck-Institut für Chemische Physik fester Stoffe, Nothnitzer Str. 40, 01277 Dresden, Germany*

Supplementary Information:

Table S1. *Crystallographic data for* $LaHg_{6.4}$

Composition	$La_8Hg_{51.2}$
Space group	*Cmcm*
Formula units per unit cell, *Z*	8
Lattice parameters	
a / Ǻ	9.779(2)
b / Ǻ	28.891(4)
c / Ǻ	5.0012(8)
V / Ǻ^3	1413.0(4)
Calc. density / g cm^{-3}	13.44
Crystal form	needle-like
Crystal size / μm	35 × 60 × 250
Diffraction system	RIGAKU AFC7
Detector	Saturn 724+ CCD
Radiation; λ / Ǻ	Mo$K\alpha$; 0.71073
Scan; step / degree; *N*(images)	φ; 0.6; 600
Maximal 2θ / degree	60.0
Range in *h*, *k*, *l*	$-13 \le h \le 13$
	$-40 \le k \le 40$
	$-3 \le l \le 6$
Absorption correction	multi-scan + numerical
T(max)/*T*(min)	9.1
Absorption coeff. / mm^{-1}	146.8
N(*hkl*) measured	5554
N(*hkl*) unique	1384
R_{int}	0.041
N(*hkl*) observed	1271
Observation criteria	$F(hkl) \ge 4\sigma(F)$
Refined parameters	59
R_F	0.063
R_w	0.065
Residual peaks / e Ǻ$^{-3}$	–0.98/1.18

*Corresponding author.
†E-mail: svanidze@cpfs.mpg.de

Table S2. *Atomic coordinates and equivalent (isotropic) displacement parameters (in Å^2) in the crystal structure of* $LaHg_{6.4}$

Atom	Site	Occupancy	*x*/*a*	*y*/*b*	*z*/*c*	$U_{iso/eq}$
La1	4c		0	0.4224(1)	¼	0.0168(7)
La2	4*c*		0	0.7247(1)	¼	0.0194(8)
Hg1	8*m*		0.1737(2)	0.62590(5)	¼	0.0235(4)
Hg2	8*m*		0.1701(2)	0.52512(5)	¼	0.0246(4)
Hg3	8*m*		0.8162(2)	0.31879(6)	¼	0.0260(5)
Hg4	8*m*		0.3469(2)	0.71438(6)	¼	0.0258(5)
Hg5*	8*f*	0.5	½	0.3363(1)	0.2845(8)	0.0262(9)
Hg6	8*m*		0.3309(2)	0.42591(7)	¼	0.0346(6)
Hg7*	4*c*	0.49(6)	0	0.103(2)	¼	0.031(2)
Hg8*	8*f*	0.14(4)	0	0.114(1)	0.280(4)	0.025(3)
Hg9*	8*f*	0.10(5)	0	0.091(3)	0.27(2)	0.042(5)
Hg10**	8*f*	0.191(7)	0	0.0105(3)	0.312(2)	0.031(2)
Hg11**	8*f*	0.190(7)	0	0.0037(4)	0.438(3)	0.033(2)

* split Hg5 and partially occupied Hg7–Hg9 sites describe local disorder in the channel wall;

**Hg10 and Hg11 sites are located within channels around [0 0 *z*] and [½ ½ *z*] axes.

Table S3. *Anisotropic displacement parameters (in Å^2) of the* $LaHg_{6.4}$ *crystal structure*

Atom	U_{11}	U_{22}	U_{33}	U_{12}	U_{13}	U_{23}
La1	0.0019(9)	0.0231(13)	0.025(2)	0	0	0
La2	0.0077(10)	0.0232(13)	0.027(2)	0	0	0
Hg1	0.0176(6)	0.0217(6)	0.0312(9)	−0.0028(5)	0	0
Hg2	0.0218(7)	0.0221(6)	0.0299(9)	0.0008(5)	0	0
Hg3	0.0211(7)	0.0293(7)	0.0275(9)	0.0022(5)	0	0
Hg4	0.0107(6)	0.0318(8)	0.0347(9)	−0.0073(5)	0	0
Hg5	0.0220(10)	0.0267(11)	0.030(2)	0	0	0.0028(14)
Hg6	0.0061(6)	0.0405(9)	0.0573(13)	0.0027(5)	0	0

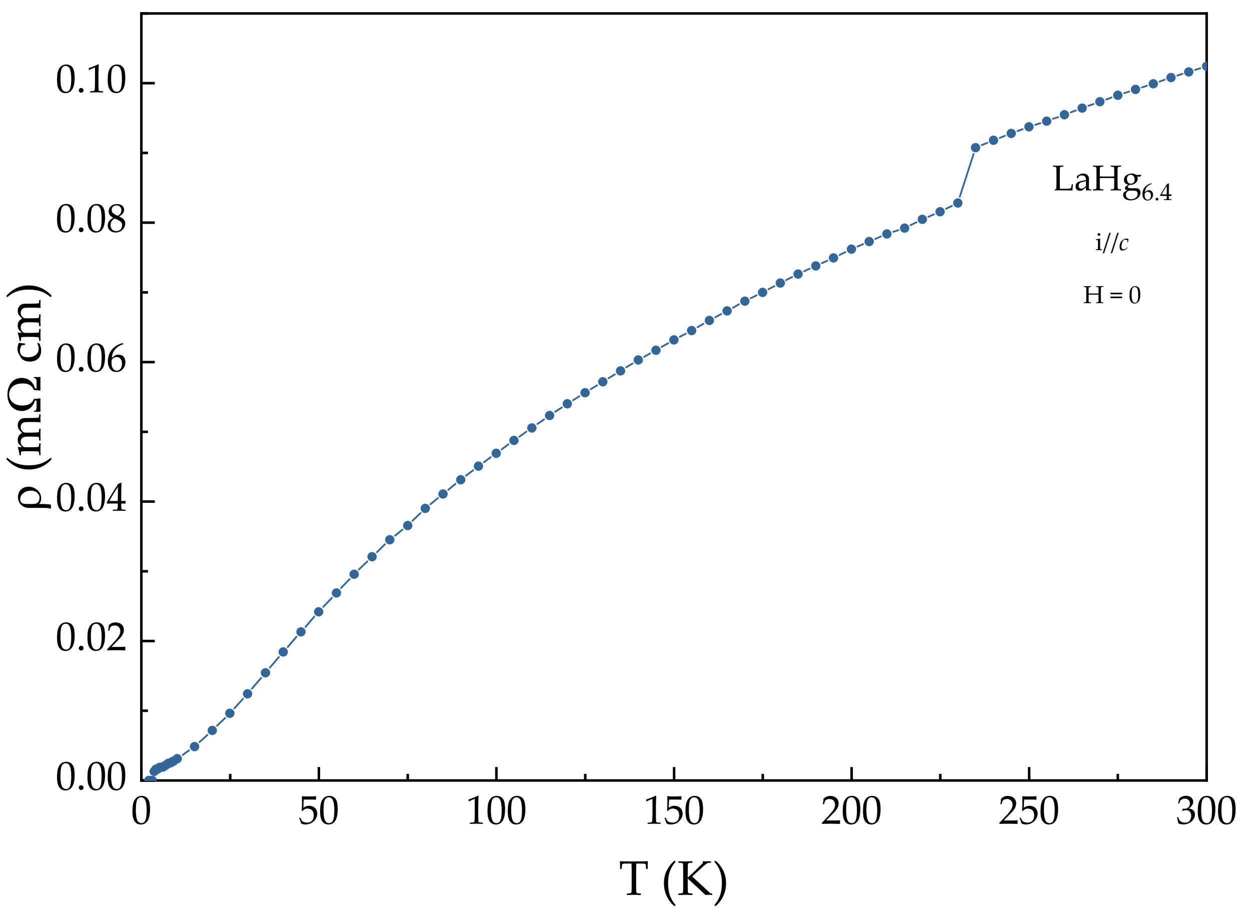

Figure S1: Temperature-dependent electrical resistivity of $LaHg_{6.4}$ single crystal with i//c in H = 0. At low-temperatures, an entrance into superconducting state is marked by a drop in resistivity to zero. At higher temperature, metallic behavior is observed, with a transition around T = 230 K, which corresponds to the freezing of elemental mercury. A residual resistivity ratio (RRR) of 63 signals good samples quality.
